# Complications of Surgically Assisted Rapid Maxillary/Palatal Expansion (SARME/SARPE)—A Retrospective Analysis of 185 Cases Treated at a Single Center

**DOI:** 10.3390/jcm13072053

**Published:** 2024-04-02

**Authors:** Rafał Nowak, Szymon Przywitowski, Paweł Golusiński, Anna Olejnik, Ewa Zawiślak

**Affiliations:** 1 Department of Otolaryngology and Maxillofacial Surgery, Institute of Medical Science, University of Zielona Góra, 65-046 Zielona Góra, Poland; 2 Face Surgery and Aesthetic Center, Pl. Powstańców Śląskich 1, 53-329 Wrocław, Poland

**Keywords:** surgically assisted maxillary/palatal expansion (SARME/SARPE), transpalatal distraction (TPD), complications, retrospective analysis

## Abstract

**Objectives:** The study aims to assess and classify complications in patients treated for maxillary transverse deficiency using surgically assisted rapid maxillary/palatal expansion (SARME/SARPE) under general anesthesia. The classification of the complications aimed to assess the difficulty of their treatment as well as estimate its real cost. **Methods**: The retrospective study covered 185 patients who underwent surgery for a skeletal deformity in the form of maxillary constriction or in which maxillary constriction was one of its components treated by a team of maxillofacial surgeons at one center (97 females and 88 males, aged 15 to 47 years, mean age 26.1 years). Complications were divided into two groups: early complications (up to 3 weeks after surgery) and late complications (>3 weeks after surgery). In relation to the occurrence of complications, we analyzed the demographic characteristics of the group, type of skeletal deformity (class I, II, III), presence of open bite and asymmetry, surgical technique, type and size of appliance used for maxillary expansion, as well as the duration of surgery. **Results**: In the study group, complications were found in 18 patients (9.73%). Early complications were found in nine patients, while late complications were also found in nine patients. Early complications include no possibility of distraction, palatal mucosa necrosis, perforation of the maxillary alveolar process caused by the distractor and asymmetric distraction. Late complications include maxillary incisor root resorption, no bone formation in the distraction gap, and maxillary incisor necrosis. None of the patients required prolonged hospitalization and only one required reoperation. **Conclusions**: Complications were found in 18 patients (9.73%). All challenges were classified as minor difficulties since they did not suppress the final outcome of the treatment of skeletal malocclusion. However, the complications that did occur required additional corrective measures. Surgically assisted rapid maxillary expansion, when performed properly and in correlation with the correct orthodontic treatment protocol, is an effective and predictable technique for treating maxillary constriction.

## 1. Introduction

A standard treatment for maxillary constriction of skeletal etiology is surgically assisted rapid maxillary expansion [1,2,3]. After bone growth is complete, orthodontic measures to increase the maxillary bone base are largely limited. The use of orthodontic forces on the teeth or hard palate to rupture the palatal suture can lead to numerous complications [4,5]. Osteotomies and released bone resistance sites in the midface allow the maxillary transverse dimension to be increased without bone resistance. This is achieved by the application of appropriate forces generated by either orthodontic appliances or distractors [6,7]. The biological phenomenon of distraction osteogenesis used in this process was first described by Codiville in 1905. In the mid-twentieth century, bone distraction was popularized and modernized by the Soviet orthopedist Ilizarov [8].

In 1999, Maurice Mommaerts introduced an alternative to tooth-borne appliances for orthognathic surgery and surgically assisted rapid maxillary expansion. This alternative came in the form of a bone-borne palatal distractor, along with his own algorithm and protocol for maxillary constriction treatment [2].

Postoperative complications are a common occurrence following surgical treatment in all medical specialties [9]. The estimation of treatment costs, and the increase in public awareness as well as the medical needs of the population, are all integral components of healthcare planning at various levels. Statistical knowledge of specific techniques is sought to measure the quality of specialist treatment. This is carried out by analyzing the number, type, and consequences of complications. Standardizing complications in surgical treatment is challenging due to significant differences in surgical procedures [10,11] and the lack of a standard definition [9]. In 2008 Dindo and Cavien defined a complication as “any deviation from the ideal postoperative course that is not inherent in the procedure and does not comprise a failure to cure” [12].

As SARME is considered a relatively safe procedure, data concerning complications are limited [13,14,15,16,17,18,19]. With a comprehensive analysis of complications, including their classification and treatment difficulty assessment, we provide valuable information that can inform clinical decision-making and enhance patient care. The unique aspect of our research lies in the substantial study group comprising 185 patients, allowing for a detailed categorization of complications and a deeper understanding of their impact on treatment outcomes.

The primary objective of this study is to systematically evaluate and classify the complications associated with SARME/SARPE in a retrospective analysis of 185 cases treated at a single center. With a particular focus on distinguishing between early complications and late complications.

The secondary objective is to evaluate the impact of patient demographics, surgical technique variations, and adjunctive therapies on the occurrence of complications associated with SARME/SARPE.

By categorizing these complications and evaluating their treatment difficulty and associated costs, we aim to provide valuable insights that can inform clinical practice and enhance patient care.

Our null hypothesis is that surgically assisted rapid maxillary expansion, when performed properly and in correlation with the correct orthodontic treatment protocol, is an effective and predictable technique for treating maxillary constriction.

## 2. Materials and Methods

The present research study was conducted in accordance with the regulations of the University of Zielona Góra, which stipulate that retrospective scientific papers, such as the one presented in our study, do not require the opinion or approval of the bioethics committee. Therefore, while we acknowledge the importance of ethical considerations in research, we followed the established guidelines of our institution for this particular type of study. The study complies with the World Medical Association Declaration of Helsinki on medical research protocols and ethics.

### 2.1. Patients

The retrospective study covered a group of 185 patients (97 females and 88 males) aged 15 to 47 years (Mean = 26.1, SD = 6.8) with a skeletal deformity in the form of maxillary constriction. The detailed age and sex distribution in the study group are shown in Table 1.

In 38 patients (20.54%), maxillary constriction and skeletal crossbite were isolated defects and surgically assisted rapid maxillary expansion was the only surgical operation used for their treatment. Other patients required more than one cosmetic procedure in the facial skeleton due to skeletal abnormalities in other planes.

Surgical treatment was carried out by a team comprising two experienced maxillofacial surgeons with extensive expertise in SARME/SARPE procedures. The surgeries were performed between January 2014 and December 2020 at the Department of Otolaryngology and Maxillofacial Surgery of the University Hospital in Zielona Góra, Poland. Surgical examinations were performed in outpatient facilities and patients were treated orthodontically in various practices throughout Poland cooperating with a team of surgeons.

The study included patients diagnosed with transverse maxillary hypoplasia on the basis of clinical and radiographic examination with all or some of the clinical symptoms of the defect, i.e., width disproportion between the upper and lower dental arches, total unilateral or bilateral crossbite, impactions in the anterior part of the maxilla, a V-shaped palate, and dark buccal corridors during smiling.

Patients with skeletal deformities resulting from craniofacial syndromes, with a history of trauma to the facial skeleton, cleft patients, and patients with incomplete medical records were excluded from the study.

The following variables were analyzed in the study: 1—age at the time of surgery, 2—sex, 3—type of skeletal deformity, 4—presence of open bite (OB), 5—presence of asymmetry (A), 6—duration of surgery in minutes, 7—type and size of distractor, 8—maxillary osteotomy with and without pterygomaxillary disjunction, 9—occurrence of postoperative complications.

Complications were classified as early, occurring up to 3 weeks after surgery (until the end of active distraction), and late—all that were found after this period.

### 2.2. Protocol of Surgical Treatment and Postoperative Examinations

Qualification for the surgical procedure was based on clinical examination and medical imaging. Medical imaging included pantomographic X-rays and/or CBCT of the skull. In addition, extraoral images of the face and occlusion, dental arch scans, and STCA cephalometric analysis were performed using NemoStudio software (NEMOTEC, Madrid, Spain). All imaging manipulation and analysis were performed by a single investigator (A.O.) who routinely performs virtual surgical planning for orthognathic surgery.

The surgical operation was performed under general anesthesia with orotracheal intubation and local anesthesia (lignocaine hydrochloride 2% + noradrenaline 0.00125% 4 mL) and perioperative antibiotic prophylaxis (Tarfazolinum iv.). In addition, patients were given Dexaven 8 mg intraoperatively and postoperatively and antibiotic therapy was extended to 5 days postoperatively (Augmentin 1 g or Zinnat 0.5 g).

**Figure 1:**
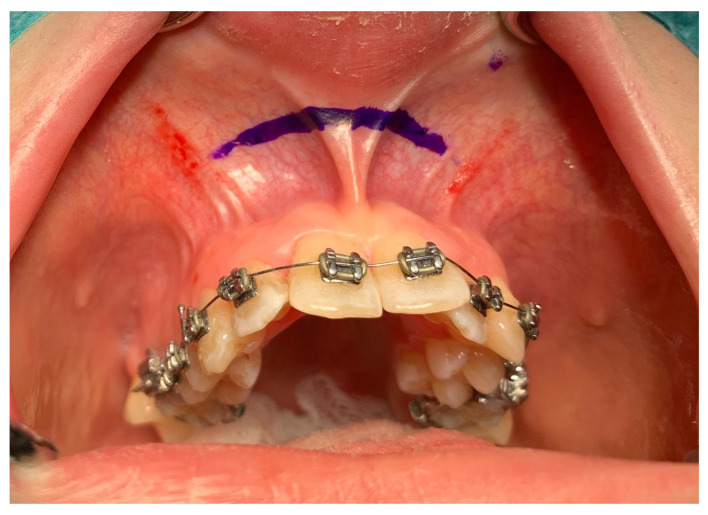
Surgical access.

**Figure 2:**
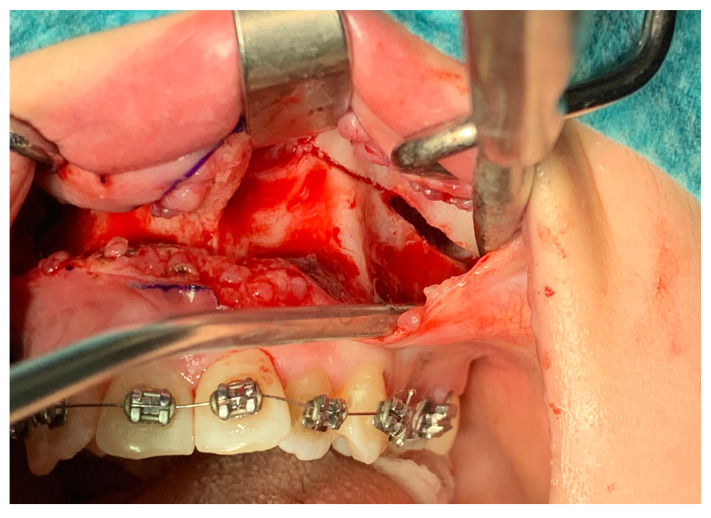
Maxillary osteotomy line—visible widening of the line with a bur.

**Figure 3:**
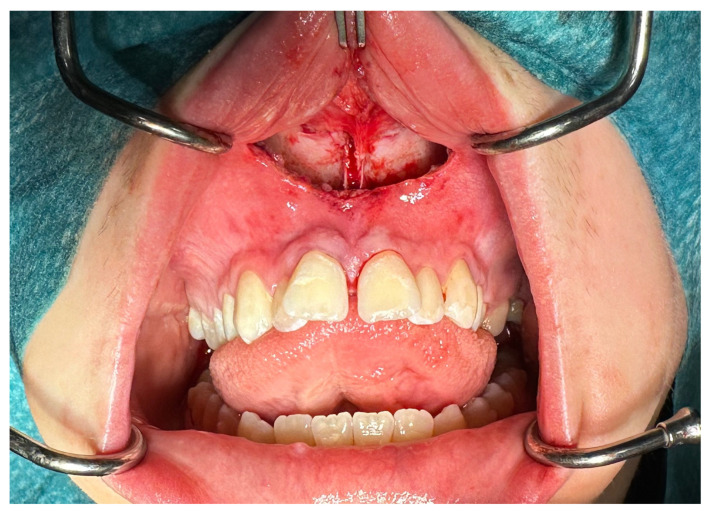
Palatal suture split.

**Figure 4:**
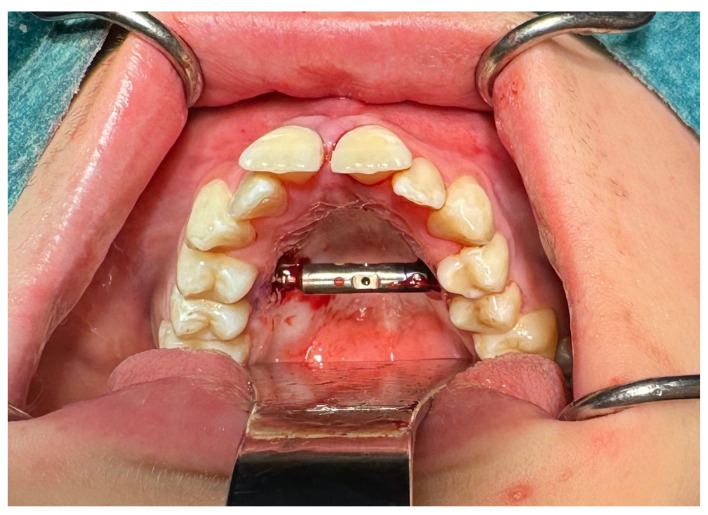
Distractor in place.

From the access in the vestibule of the oral cavity (Figure 1), using a piezoelectric saw, a maxillary osteotomy was performed along the Le Fort I line. In addition, the osteotomy line in the area of the premolars and molars was widened with a 2 mm carbide round bur (Figure 2); then the palatal suture was split using a piezoelectric saw and chisels (Figure 3). In some cases, the maxilla was separated from the pterygoid processes of the sphenoid bone (pterygomaxillary junction, PMJ) [2,20,21,22]. The decision to separate the maxilla from the sphenoid bone was determined by the original shape of the dental arch [23]. No separation was made in the pterygomaxillary junction to accommodate the need for expansion in the anterior region of the dental arch. In each case, the mobility of the maxillary fragments was checked prior to distractor placement. A palatal distractor was then placed in a typical location on the palate (in 177 cases, Figure 4), except for eight cases of patients who had a tooth-borne hyrax appliance installed prior to surgery.

The distractor was activated mid-operatively until a 1.0 mm diastema appeared between the central incisors. Two similar distractors (Titamed, Kontich, Belgium, n = 169; ChM, Lewickie, Poland, n = 8) and orthodontic expanders with a hyrax screw (n = 8) were used. As with the hyrax screw expanders, the distractors often lacked a replaceable transversal module. The distractor was then locked. Hemostasis was controlled, and the wound was closed with 4.0 absorbable sutures.

The postoperative latency period ranged from 5 to 7 days. At the first postoperative outpatient appointment, after radiographic examination (OPG), the distractor was activated, and the active maxillary expansion phase began. The duration of the expansion phase depended individually and ranged from 10 to 28 days, with an average of 18 days. Once a day, the module was activated by 0.25 mm (Titamed, Kontich, Belgium) or 0.35 mm (ChM, Lewickie, Poland). Standard examinations were conducted at 7-day intervals during the distraction period.

After determining the intended width of the upper arch, the distractor was locked, thus concluding the active phase of distraction.

Active orthodontic treatment and diastema closure were started 6–8 weeks after distractor closure [24]. After the closure of the distractor, it was recommended that a NiTi 0.16 × 022 orthodontic arch be placed. Gradual closure of the diastema, stabilization of the lateral sections, and individual orthodontic treatment followed.

The consolidation period lasted on average 6 months from the end of active distraction, after which the distractor was removed under local anesthesia [25,26].

In patients treated with the hyrax device, the surgical part differed only at the stage of distractor placement—which did not occur in these cases—while the postoperative treatment followed the same protocol.

The complications were found at the stage of surgical examinations as well as conservative management and orthodontic treatment.

### 2.3. Methods of Statistical Analysis

The Mann–Whitney test was used to compare the duration of surgery (quantitative variable) between the two groups, and the Kruskal–Wallis test (followed by the post-hoc Dunn test) was used for three or more groups.

The chi-squared test (with Yates correction for 2 × 2 tables) or the Fisher’s exact test (when low values were expected) was used to compare qualitative variables (e.g., other than the duration of surgery) between the groups.

The significance level was set at 0.05.

All analyses were performed using R software, version 4.3.2.

## 3. Results

In the study group, the proportions between females and males were not statistically different—52.43% vs. 47.57%. No statistically significant relationship was found between the sex and specific age groups (*p* > 0.05).

Based on the cephalometric analysis, three types of skeletal malocclusion (skeletal class I, II, III), asymmetry (A), and open bite (OB) were distinguished in the study group. The sex distribution in relation to the skeletal malocclusion type and the occurrence of asymmetry (A) and open bite (OB) in relation to the skeletal malocclusion type were analyzed. A statistically higher percentage of females was found in patients with skeletal class II (63.24%) and the lowest in patients with skeletal class III (40.51%, *p* = 0.017).

Open bite (OB) was found in 23.08% (n = 39) of patients and asymmetry in 15.68% (n = 29) of the study group.

The percentage of patients with open bite (OB) was highest in class II patients (36.76%) and lowest in class III patients (10.13%, *p* < 0.001). The percentage of patients with asymmetry (A) was highest in class I patients (39.46%) and lowest in class II patients (7.35%, *p* < 0.001).

**Figure 5:**
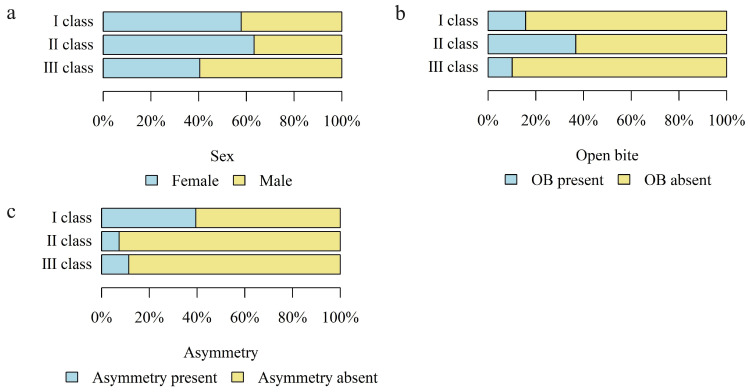
The number (percentage) of patients in the groups with different skeletal classes and the (**a**) sex distribution; (**b**) presence of open bite (OB); (**c**) presence of asymmetry.

Figure 5 shows a detailed distribution of the skeletal class related to the above-mentioned characteristics.

The mean duration of surgery in minutes in the study group was 79.63 (SD = 24.05, median = 75.0). The longest treatment lasted 180 min, and the shortest 30 min (Q1 = 65, Q3 = 90).

The duration of surgery in minutes was analyzed in relation to skeletal malocclusion, presence of open bite (OB), and asymmetry (A).

The duration of surgery was significantly longer in patients without asymmetry than in those with asymmetry.

**Figure 6:**
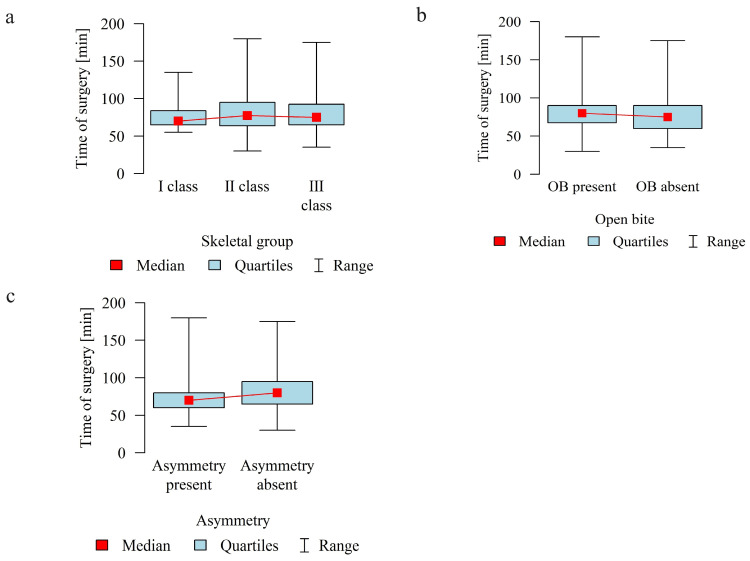
Duration of surgery in minutes according to the (**a**) skeletal class (I, II, III); (**b**) presence of open bite (OB); (**c**) presence of asymmetry.

Table 2 and Figure 6 show details of the analysis of the duration of surgery in relation to the above variables.

**Figure 7:**
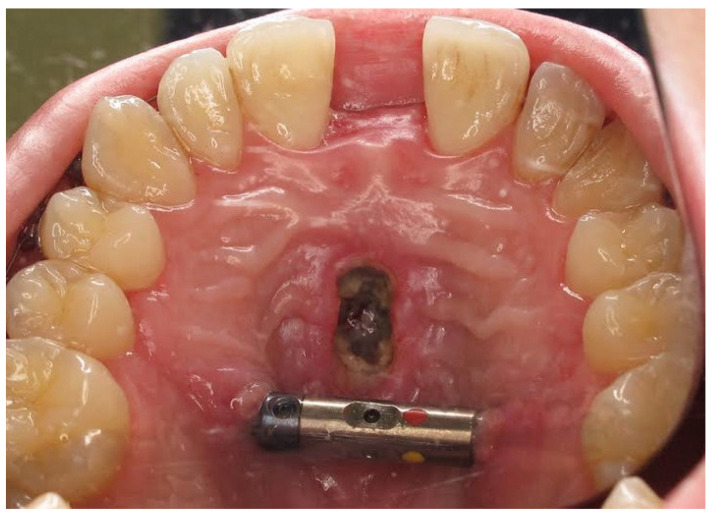
Palatal mucosal necrosis.

**Figure 8:**
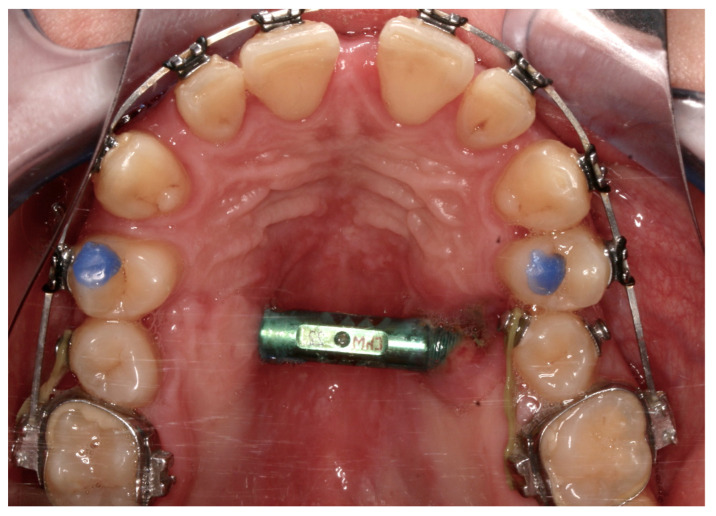
Perforation of the maxillary alveolar process caused by the distractor.

**Figure 9:**
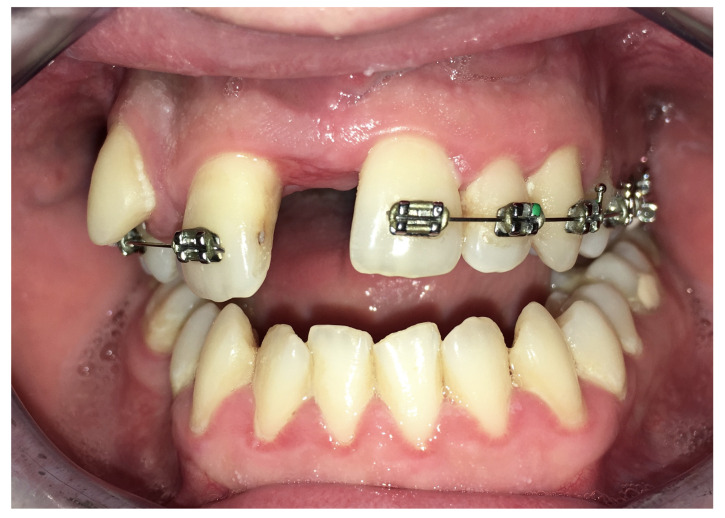
Asymmetric distraction.

**Figure 10:**
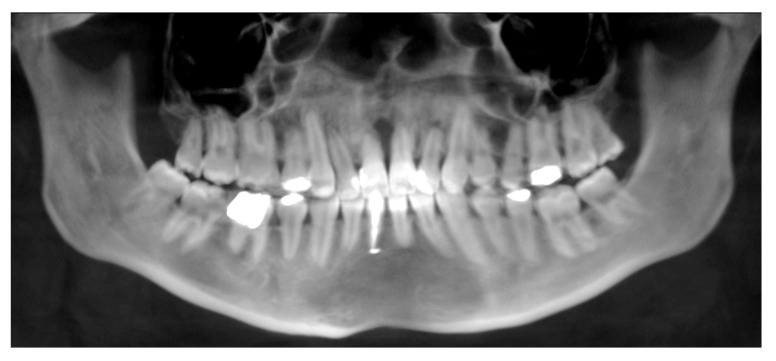
Root resorption of the central and lateral incisors.

**Figure 11:**
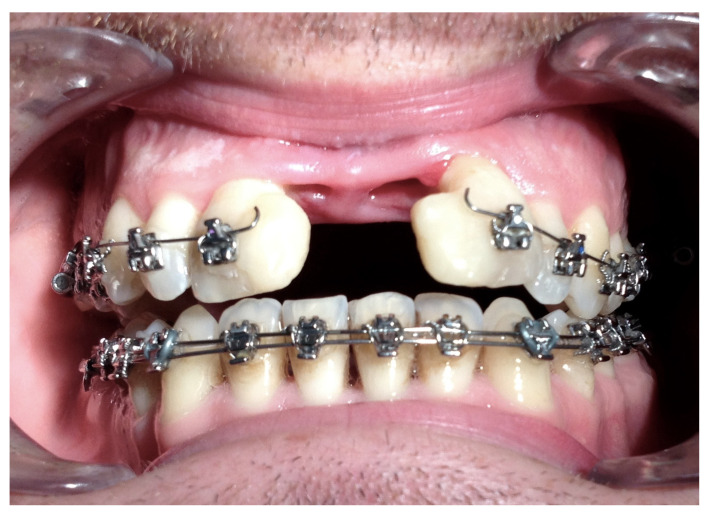
Bone loss/lack of bone formation in the distraction gap.

**Figure 12:**
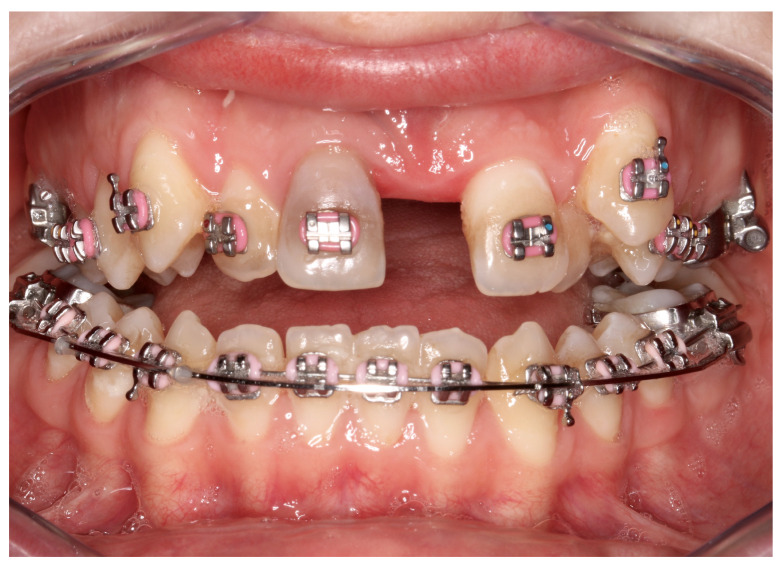
Necrosis of tooth 11.

Complications were found in 18 patients (9.73%) of the study group—Table 3. Early complications (up to 3 weeks after surgery) were found in nine patients, and late complications in the same number of patients (nine patients). Early complications include no possibility of distraction—1 case, palatal mucosal necrosis—2 cases, perforation of the maxillary alveolar process caused by the distractor—1 case, and asymmetric distraction—5 cases (Figure 7, Figure 8 and Figure 9). Late complications include maxillary incisor root resorption—2 cases, bone loss/lack of bone formation in the distraction gap—5 cases, and maxillary incisor necrosis—2 cases (Figure 10, Figure 11 and Figure 12). All were observed during the post-operative treatment period.

One case of early complication (no possibility for expansion) required re-operation. It was probably caused by a blockage of the maxillary bone fragments or their insufficient surgical release.

The occurrence of complications was analyzed according to age, sex, skeletal class, presence of open bite and asymmetry, separation in the pterygomaxillary junction, type and size of the distractor, and duration of surgery in minutes in the study group.

There was no statistically significant correlation between the occurrence of complications and age, sex, presence of asymmetry (A), separation in the PMJ, type and size of the distractor, and the duration of surgery in minutes.

A statistically significant higher percentage of complications was found in patients with skeletal class II and the lowest in patients with skeletal class I.

**Figure 13:**
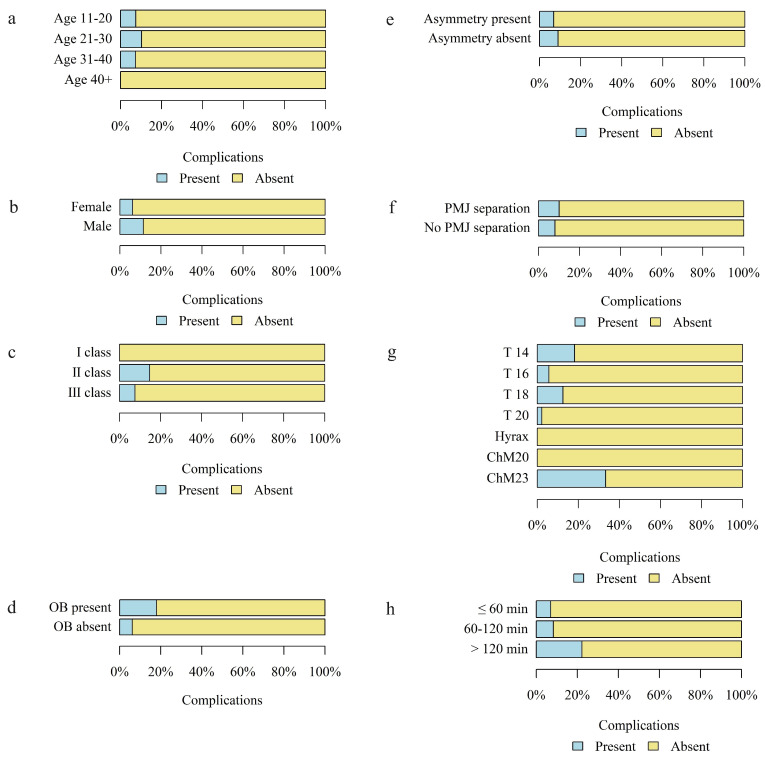
The occurrence of complications according to the (**a**) age group of patients; (**b**) sex; (**c**) skeletal class; (**d**) occurrence of open bite (OB); (**e**) occurrence of asymmetry; (**f**) separation in the PMJ; (**g**) type and size of distractor; (**h**) duration of surgery in minutes.

Details of the above analysis are presented in Table 4 and Figure 13.

## 4. Discussion

Surgically assisted rapid maxillary expansion is an effective and popular technique for treating complete crossbite after bone growth is complete or when orthodontic methods have failed [2,3,23]. The technique, which uses the phenomenon of distraction osteogenesis, enables increasing the bone base of the maxilla with simultaneous lengthening of the soft tissues. This is particularly important in the region of the less susceptible mucosa of the hard palate [27].

The aim of this study was to evaluate and classify complications of surgically assisted maxillary expansion in order to assess the safety of the technique as well as the real cost of treatment. It is important for both orthodontists and patients to be aware of the complications of orthodontic and surgical team treatment. Apart from the occurrence of complications, we analyzed the demographic characteristics of the group, type of skeletal deformity (classes I, II, III), presence of open bite and asymmetry, surgical technique, type and size of the distractor, as well as the duration of surgery.

There was no statistically significant correlation between the occurrence of complications and age, sex, presence of asymmetry (A), separation in the PMJ, type and size of the distractor, and the duration of surgery in minutes. We only found a statistically significant higher percentage of complications in patients with skeletal class II and the lowest in patients with skeletal class I.

In the available medical literature, no data were found on complications of SARPE according to the skeletal class. The only analyses available in the literature are on the distribution of complications according to the skeletal class in orthognathic surgery, excluding SARPE [28,29].

Serious complications resulting from uncontrolled fractures of the facial skeleton and cranial base and damage to vascular and neural structures as a result of SARPE are rarely reported [19,30,31,32]. It is now known that a maxillary incision along the Le Fort I line and separation at the palatal suture results in expansion without negative effects on distant structures of the facial skeleton and neurocranium [33,34].

In the study group of 185 patients, we found complications in 18 cases (9.73%).

Early complications, up to 3 weeks after surgery, were found in nine patients (4.86%). One of the complications required reoperation (0.54%). There were no complications requiring urgent surgical intervention and no cases of prolonged hospitalization.

Asymmetric distraction (n = 5; 2.7%) was corrected during orthodontic treatment and/or bimaxillary osteotomies or Le Fort I osteotomies. Mucosal necrosis (n = 2; 1.08%) and maxillary bone perforation by the distractor (n = 1; 0.54%) were managed conservatively without negative consequences on the final treatment outcome.

Late complications, found up to three weeks after surgery, were found in nine patients (4.86%). All late complications were related to the maxillary central incisors, which may be related to the separation of the palatal suture [14,35].

Root resorption of the maxillary central incisors is one of the most common complications of orthodontic treatment [36,37]. The question remains to what extent this complication is related to surgical treatment and to what extent to orthodontic therapy. Incisor root resorptions (n = 2; 1.08%) were left for observation and maxillary incisor necrosis (n = 2; 1.08%) was managed conservatively.

In relative terms, the greatest difficulty was the lack of adhesion between the maxillary incisors (n = 5; 2.7%). This complication was managed conservatively by prolonged retention of the orthodontic appliance without its activation [36,37]. In two cases, bone grafting was performed under local anesthesia in the area where no adhesion was found, and in one case bone grafting was performed at the stage of the Le Fort I osteotomy. In addition, these patients underwent corrections to the shape of the central incisors. There was no loss of maxillary incisors in any of the late complications.

Complications in the group of patients treated by our team were compared with the study by Verquin et al. and a much lower percentage was found in our study (9.73% vs. 50.9%) [18].

The authors report the occurrence of complications in 28 patients (50.9%) in the study group of 55 patients, i.e., about half of the group treated with SARPE. These were complications in the form of postoperative hemorrhage in six patients (10.9%), infraorbital nerve damage (n = 16; 29.1%), dental complications (n = 5; 9.1%), problems with the appliance (n = 3; 5.5%), asymmetric expansion (n = 3; 5.5%), severe postoperative pain (n = 4; 7.3%), and other complications (n = 7; 12.7%). Six patients (10.9%) required prolonged hospitalization and two (3.63%) required additional surgery. The occurrence of hemorrhage, infraorbital nerve damage, or severe postoperative pain was not reported by our study. During the postoperative period, we found temporary sensory disturbances in the nervation of the infraorbital nerve. However, they did not become permanent, and we did not consider them to be a complication. No patient required prolonged hospitalization. One patient required reoperation due to a lack of distraction progression, which represents 0.54% of the studied group. Compared to the study by Verquin et al., the need for reoperation was lower in our study (3.63% vs. 0.54%) [18].

Our study shows the occurrence of dental complications and asymmetric distraction, similar to the study by Verquin et al. [18]. Dental complications in our study, such as incisor root resorption, lack of adhesion between incisors (incisor mobility), and incisor necrosis, totaled nine cases (4.86%). This statistic is comparable to the one found in the study conducted by the abovementioned authors. The researchers report that dental complications occurred in 5.5% of the group they studied.

Asymmetric distraction was found in five cases (2.7%) in our study, while it was twice as high in the study by Verquin et al., i.e., 5.5% of patients treated with SARME [18].

Williams et al. conducted a study on a group of 120 patients who underwent SARPE treatment [17]. The study found that complications occurred in 41 cases (34.16%), of which 33 cases (27.5%) were surgical complications. The authors divided the complications into surgical, dental, and periodontal. Complex complications from each group occurred in ten patients (8.33%). Surgical complications included hemorrhage and hematoma (n = 7; 5.83%), infection (n = 8; 6.66%), tissue necrosis (n = 1; 0.83%), V2 hypoesthesia (n = 4; 3.33%), asymmetric expansion (n = 10; 8.33%), insufficient expansion (n = 9; 7.5%), and a combination of asymmetric expansion and insufficient expansion (n = 3; 2.5%). Furthermore, within the group experiencing surgical-dental/periodontal complications, occurrences of hemorrhage with tooth discoloration (n = 1; 0.83%), insufficient expansion with gingival recession (n = 4; 3.33%), infection with tooth discoloration (n = 1; 0.83%), gingival recession (n = 2; 1.66%), dental bone loss (n = 3; 2.5%), and tooth loss (n = 2; 1.66%) were found. One patient experienced several complications, which were counted as one single case in a specific group. For example, V2 hypoesthesia was found in four patients, and three of them had hemorrhage, infection, and hematoma.

The total number of complications in our study is lower than in the study by Williams et al.—9.73% vs. 34.16% [17].

Our study did not report any cases of hemorrhage or hematoma, infection, V2 hypoesthesia, or insufficient expansion. As in the Williams study, tissue necrosis, asymmetric distraction, and dental complications occurred in our study group. Tissue necrosis was found in two cases (1.08%)—a similar number to the study by Williams et al. (n = 1, 0.83%) [17].

Asymmetric distraction and dental complications were at lower levels than in the study by Williams et al. (2.7% vs. 8.33% and 4.86% vs. 15.0%), but they were the most common in our study [17].

Dental complications of SARPE are a frequent topic of discussion [5]. The complications can be divided into two groups. The first is dental-periodontal complications found when tooth-borne appliances are used. The second includes dental complications related to the central maxillary incisors. These complications were found in maxillary expansion treatment, whether using tooth-borne or bone-borne expanders [38].

Complications were also found in the second group in our material. The issues related to central incisors in surgically assisted rapid maxillary expansion are the result of multiple factors [17,39,40]. Direct or indirect injury during an osteotomy of the palatal suture or distraction of the maxillary fragments may cause damage to the marginal and apical periodontium of the incisors. Impaired perfusion of the periodontal tissues is the second cause of damage. This can lead to necrosis of the dental pulp, root end resorption, and hypermobility, ultimately resulting in tooth loss. Dental complications reportedly account for up to 20% of cases in patients undergoing SARPE treatment, according to various sources [16,17,41].

Orthodontically positioning the roots of the maxillary central incisors before surgery appears to be a good solution for minimizing dental complications after surgery. Oztürk et al. reported that hypoperfusion of the maxillary central incisors reaches its peak on the third day after SARPE and is 60% of the normal level by the seventh day after surgery [42]. Activation of the distractor earlier than seven days after surgery may lead to ischemia of the maxillary central incisors and affect dental complications in this region.

The frequency of asymmetric distraction in SARPE treatment ranges from 3.4% to 18%, according to various authors [16,17,18,41,43,44].

Koudstaal et al. conducted an experimental study on skulls and found asymmetric distraction in 4 out of 10 cases [45]. The study on ten cadaveric skulls analyzed the rotation of maxilla fragments in the frontal plane after maxillary distraction using both tooth-borne and bone-borne appliances. The authors’ observation of asymmetric distraction in the case of tooth-borne and bone-borne appliances is noteworthy since it is known that the angular rotation of the bone fragments is smaller in these cases [45,46]. The conclusion of our study is that, firstly, it is important to pay close attention to the separation of the maxilla from the sphenoid bone, and, secondly, to consider the ontogenetic susceptibility of the soft tissues, such as muscles and ligaments, to distraction. Our study found that there were three cases of asymmetric distractions with simultaneous pterygomaxillary disjunction and two cases of asymmetric distractions without pterygomaxillary disjunction. This supports the claim that the ontogenetic susceptibility of soft and hard tissues has an impact on this complication.

To achieve symmetrical distraction, a full horizontal osteotomy and a palatal suture osteotomy should be performed, along with incision of the lateral nasal walls [3,15,17].

In our study, no complications related to the use of the distractor were found. This seems to be related to the construction of the distractors, where the transverse module is not a separate element. In studies where the distractor issue was observed, it was found that the screw module was a replaceable element.

In addition to assessing the difficulty of treatment, our study also considered treatment costs associated with the identified complications. While all challenges were classified as minor difficulties, they required additional corrective measures which incurred costs. One of the complications required reoperation. There were no complications requiring urgent surgical intervention and no cases of prolonged hospitalization. The other costs were primarily related to conservative dentistry procedures such as endodontic treatment, as well as oral surgery procedures like bone grafting.

There are some limitations of our study. It was conducted at a single center, which may limit the generalizability of the findings to a broader population. Multi-center studies with larger and more diverse patient cohorts would provide more robust evidence. The retrospective nature of the study could introduce bias and limit the ability to establish causal relationships. Prospective studies would offer stronger evidence. Although our study included a substantial number of patients (185 cases), a larger sample size could provide more statistical power and allow for subgroup analyses based on different variables. All imaging analyses were performed by a single investigator, which could introduce potential bias or limitations in the accuracy and reliability of measurements. Future studies should consider interobserver reliability assessments.

## 5. Conclusions

Complications were found in 18 patients (9.73%). Early complications were found in nine patients (4.86%) and late complications were found in nine patients (4.86%). All challenges were classified as minor difficulties since they did not suppress the final outcome of the treatment of skeletal malocclusion. All complications occurred in the outpatient setting and did not require urgent surgical intervention. However, the complications that did occur required additional corrective measures.

Surgically assisted rapid maxillary expansion, when performed properly and in correlation with the correct orthodontic treatment protocol, seems to be an effective and predictable technique for treating maxillary constriction.

## Figures and Tables

**Table 1:** Detailed age and sex distribution in the study group.

Sex	Age Range					*p*
	Age 11–20 (N = 40)	Age 21–30 (N = 97)	Age 31–40 (N = 41)	Age 40+ (N = 7)	Total (N = 185)	
Female	24 (60.00%)	43 (44.33%)	25 (60.98%)	5 (71.43%)	97 (52.43%)	*p* = 0.128
Male	16 (40.00%)	54 (55.67%)	16 (39.02%)	2 (28.57%)	88 (47.57%)	

*p*—chi-squared test.

**Table 2:** Detailed analysis of the duration of surgery (min) in relation to skeletal malocclusion, open bite, and asymmetry.

Parameter	Group	Duration of Surgery [min]							*p*
		Mean	SD	Median	Min	Max	Q1	Q3	
Skeletal group	I class (N = 38)	76.32	17.19	70.0	55	135	65.00	83.75	*p* = 0.695
	II class (N = 68)	81.57	26.85	77.5	30	180	63.75	95.00	
	III class (N = 79)	79.56	24.42	75.0	35	175	65.00	92.50	
Open bite	OB present (N = 39)	82.95	26.00	80.0	30	180	67.50	90.00	*p* = 0.314
	OB absent (N = 146)	78.75	23.52	75.0	35	175	60.00	90.00	
Asymmetry	Asymmetry present (N = 29)	72.07	24.62	70.0	35	180	60.00	80.00	*p* = 0.015 *
	Asymmetry absent (N = 156)	81.04	23.76	80.0	30	175	65.00	95.00	

2 groups comparison: *p*—Mann–Whitney test; >2 groups comparison: Kruskal–Wallis test + post-hoc analysis (Dunn test). * statistically significant (*p* < 0.05).

**Table 3:** Early and late complications in a group of 185 patients treated with Surgically Assisted Rapid Maxillary/Palatal Expansion (SARME/SARPE).

Early Complications/up to 3 Weeks after Surgery	Late Complications/ > 3 Weeks after Surgery
No possibility of distraction—1 case	Maxillary incisor root resorption—2 cases
Palatal mucosal necrosis—2 cases	Bone loss/lack of adhesion in the distraction gap—5 cases
Perforation of the maxillary alveolar process caused by the distractor—1 case	Maxillary incisor necrosis—2 cases
Asymmetric distraction—5 cases	
9 cases (4.86%)	9 cases (4.86%)

**Table 4:** The occurrence of complications analysis.

Parameter	Group	Complications		*p*
		Present	Absent	
Age range	Age 11–20 (N = 40)	3 (7.50%)	37 (92.50%)	*p* = 0.91
	Age 21–30 (N = 97)	10 (10.31%)	87 (89.69%)	
	Age 31–40 (N = 41)	3 (7.32%)	38 (92.68%)	
	Age 40+ (N = 7)	0 (0.00%)	7 (100.00%)	
Sex	Female (N = 97)	6 (6.19%)	91 (93.81%)	*p* = 0.322
	Male (N = 88)	10 (11.36%)	78 (88.64%)	
Skeletal group	I class (N = 38)	0 (0.00%)	38 (100.00%)	*p* = 0.023 *
	II class (N = 68)	10 (14.71%)	58 (85.29%)	
	III class (N = 79)	6 (7.59%)	73 (92.41%)	
Open bite	OB present (N = 39)	7 (17.95%)	32 (82.05%)	*p* = 0.047 *
	OB absent (N = 146)	9 (6.16%)	137 (93.84%)	
Asymmetry	Asymmetry present (N = 29)	2 (6.90%)	27 (93.10%)	*p* = 1
	Asymmetry absent (N = 156)	14 (8.97%)	142 (91.03%)	
PMJ separation	PMJ separation (N = 60)	6 (10.00%)	54 (90.00%)	*p* = 0.862
	No PMJ separation (N = 125)	10 (8.00%)	115 (92.00%)	
Distractor	T 14 (N = 22)	4 (18.18%)	18 (81.82%)	*p* = 0.06
	T 16 (N = 53)	3 (5.66%)	50 (94.34%)	
	T 18 (N = 48)	6 (12.50%)	42 (87.50%)	
	T 20 (N = 46)	1 (2.17%)	45 (97.83%)	
	Hyrax (N = 8)	0 (0.00%)	8 (100.00%)	
	ChM20 (N = 2)	0 (0.00%)	2 (100.00%)	
	ChM23 (N = 6)	2 (33.33%)	4 (66.67%)	
Duration of surgery	≤60 min (N = 43)	3 (6.98%)	40 (93.02%)	*p* = 0.31
	60–120 min (N = 133)	11 (8.27%)	122 (91.73%)	
	>120 min (N = 9)	2 (22.22%)	7 (77.78%)	

*p*—chi-squared or Fisher’s exact test. * statistically significant (*p* < 0.05).

## Data Availability

Data is contained within the article.
